# Neural Correlates of Auditory Perceptual Awareness and Release from Informational Masking Recorded Directly from Human Cortex: A Case Study

**DOI:** 10.3389/fnins.2016.00472

**Published:** 2016-10-20

**Authors:** Andrew R. Dykstra, Eric Halgren, Alexander Gutschalk, Emad N. Eskandar, Sydney S. Cash

**Affiliations:** ^1^Program in Speech and Hearing Bioscience and Technology, Harvard-MIT Division of Health Sciences and TechnologyCambridge, MA, USA; ^2^Department of Neurology, Massachusetts General Hospital and Harvard Medical SchoolBoston, MA, USA; ^3^Departments of Radiology and Neurosciences, University of CaliforniaSan Diego, La Jolla, CA, USA; ^4^Department of Neurology, Ruprecht-Karls-Universität HeidelbergHeidelberg, Germany; ^5^Department of Neurosurgery, Massachusetts General Hospital and Harvard Medical SchoolBoston, MA, USA

**Keywords:** auditory cortex, conscious perception, electrocorticography, high-gamma activity, informational masking

## Abstract

In complex acoustic environments, even salient supra-threshold sounds sometimes go unperceived, a phenomenon known as informational masking. The neural basis of informational masking (and its release) has not been well-characterized, particularly outside auditory cortex. We combined electrocorticography in a neurosurgical patient undergoing invasive epilepsy monitoring with trial-by-trial perceptual reports of isochronous target-tone streams embedded in random multi-tone maskers. Awareness of such masker-embedded target streams was associated with a focal negativity between 100 and 200 ms and high-gamma activity (HGA) between 50 and 250 ms (both in auditory cortex on the posterolateral superior temporal gyrus) as well as a broad P3b-like potential (between ~300 and 600 ms) with generators in ventrolateral frontal and lateral temporal cortex. Unperceived target tones elicited drastically reduced versions of such responses, if at all. While it remains unclear whether these responses reflect conscious perception, itself, as opposed to pre- or post-perceptual processing, the results suggest that conscious perception of target sounds in complex listening environments may engage diverse neural mechanisms in distributed brain areas.

## Introduction

In complex acoustic environments, perceiving sounds of interest is often limited by information-processing bottlenecks in the central auditory system rather than resolution of the auditory periphery, a phenomenon known as informational masking (Pollack, 1975; Kidd et al., 2008). How the brain overcomes informational masking and gates target sounds to perceptual awareness is not well-understood (but see Gutschalk and Dykstra, 2014) despite very early studies revealing neural correlates of auditory perceptual awareness for simple acoustic scenes (Hillyard et al., 1971; Squires et al., 1973). Furthermore, beyond shedding light on how the auditory system supports auditory perceptual awareness and release from informational masking, such studies can also inform the study of conscious perception across sensory modalities (Cariani and Micheyl, 2012; Snyder et al., 2015), for which there is a paucity of data outside the context of vision (Dehaene and Changeux, 2011; Koch et al., 2016).

A commonly-used paradigm to study informational masking involves presenting a target stream of tones amidst a random multi-tone background (Neff and Green, 1987), with target tones surrounded by a protected frequency region (Neff et al., 1993) to prevent energetic masking at the auditory periphery (Delgutte, 1990). Such randomly-varying maskers, combined with uncertainty about the features comprising the target (e.g., if the pitch of the target varies across trials), drastically decrease the probability of target sounds reaching awareness (Kidd et al., 2008). Recent neurophysiological studies have suggested a role for non-primary auditory cortex in perception of Gutschalk et al. (2008) or selective attention to Elhilali et al. (2009) a rhythmic target stream amidst complex maskers (for review see Gutschalk and Dykstra, 2014). However, what role other brain areas might play in overcoming informational masking and gating target sounds to awareness remains an open question.

The present study combined intracranial EEG (iEEG) recordings in a neurosurgical patient with an auditory target-detection task in order to further characterize the neural correlates of auditory perceptual awareness under informational masking. We asked whether such correlates extend either into brain areas outside auditory cortex or into high-gamma activity (HGA), high-frequency local field potentials (LFP) thought to reflect a combination of multi-unit firing and high-frequency synaptic activity (Steinschneider et al., 2008; Manning et al., 2009; Lachaux et al., 2012).

Due to its high resolution in both space and time, proximity to potential generators, and reasonably broad coverage (particularly over peri-Sylvian areas), iEEG is well-positioned to address these questions. HGA, in particular, is challenging to observe with non-invasive methods such as EEG or MEG (Lachaux et al., 2012). The fMRI BOLD signal correlates well with HGA (particularly when compared with other LFP frequencies; Mukamel et al., 2005), but is extremely slow (on the order of seconds) compared to HGA dynamics, which unfold on the order of tens of ms. With regard to extra-auditory activity, although MEG and EEG have broader coverage than typical iEEG recordings, MEG and EEG see only that brain activity which propagates to the scalp, which is only a small fraction of true source activity due to cancelation effects (Ahlfors et al., 2010; Irimia et al., 2012). Furthermore, source estimation of activity that can be seen with M/EEG is inherently uncertain, particularly for distributed sources that are not known *a priori*, and thus requires confirmatory evidence from inside the head (Halgren, 2004).

**Figure 1:**
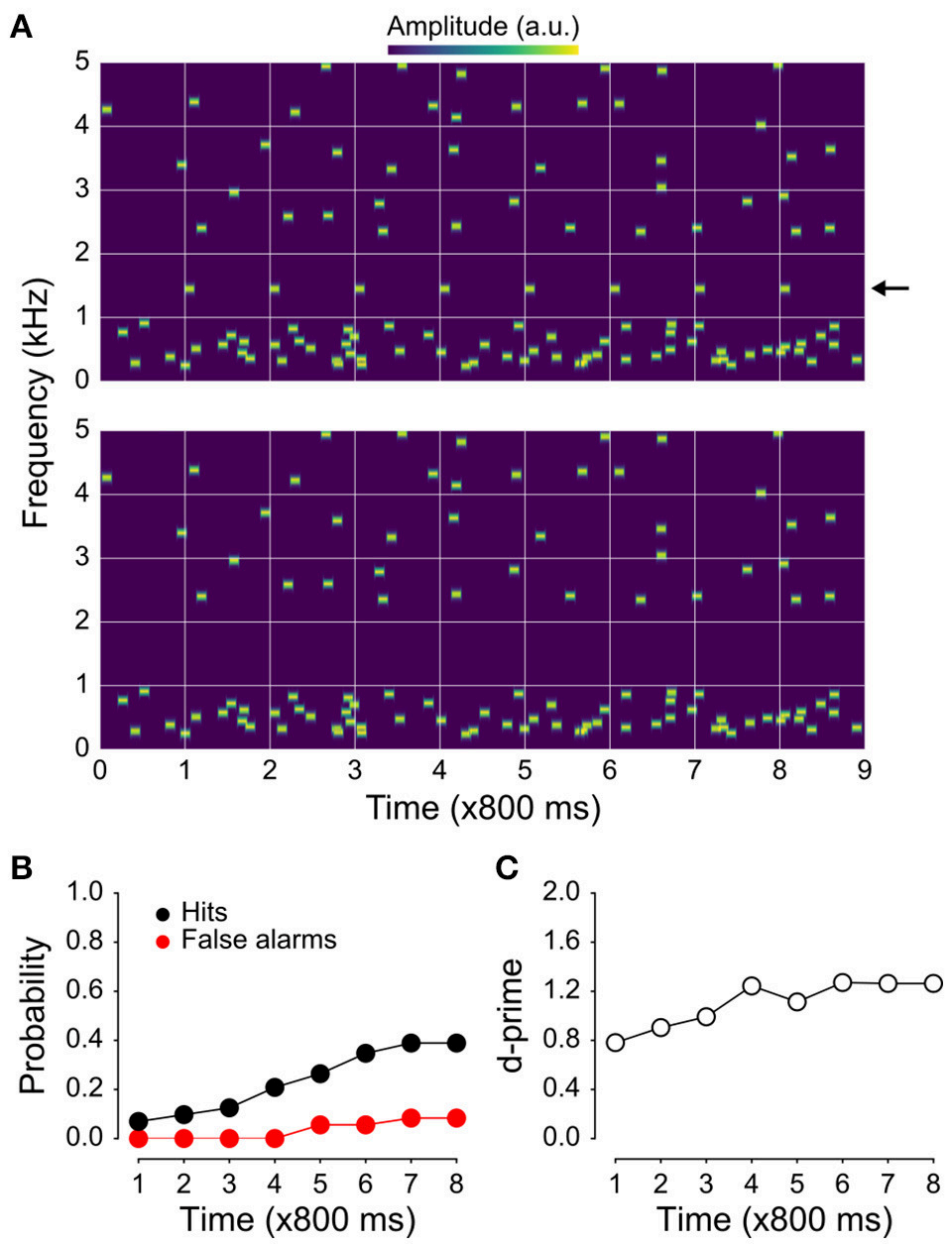
**Example stimuli and behavioral results**. **(A)** Spectrogram of the jittered multi-tone masker stimulus used in the present study with regularly repeating target tones at 1 kHz (top), and the same stimulus with the targets absent (bottom). **(B)** Hit and false-alarm rates. The false-alarm rate across time since sequence onset is shown in red; hit rate in black. Although the target tones are easily segregated visually in the spectrograms shown in **(A)**, they were not as easy to detect. Hit rates were substantially higher than false-alarm rates, resulting in target-sensitivity (d′) values **(C)** greater than one by the 4th position in the stimulus sequence. The fact that d′ values are initially rather high after only one target-tone presentation likely reflects the nature of the task (i.e., that two target tones prior to the awareness-indicating button press are included in the “detected” bin and counted as hits).

The patient, who was undergoing invasive epilepsy monitoring, listened to sequences of random masker tones that sometimes contained an isochronous target stream (Figure 1A; cf. Supplemental Audio Files 1–4) which, due to informational masking, was only sometimes detected. Targets that were detected elicited focal early activity (including both HGA and a robust negativity between 100 and 200 ms) in posterolateral auditory cortex that was diminished or absent for undetected targets as well as a broad, long-latency response, that spread to ventrolateral frontal and lateral temporal cortices. The results suggest that detecting sounds of interest in adverse listening situations may engage diverse brain areas, including auditory cortex on the posterior superior temporal gyrus (*p*STG) as well as frontal and temporal areas involved in attention and target detection (Halgren, 2008).

## Materials and methods

### Ethics statement

All procedures were approved by the Institutional Review Boards at Partners Healthcare (Massachusetts General Hospital) and the Massachusetts Institute of Technology (MIT) in accordance with NIH guidelines. Written informed consent was obtained from all patients prior to their participation. The research had no impact on the clinical care of the patients.

### Listeners

Five patients with intractable epilepsy undergoing invasive monitoring for localization of epileptogenic foci participated in the study. Each patient was implanted with sub-dural platinum electrodes embedded in silastic (2.3 mm exposed diameter, 10 mm center-to-center spacing; Ad-tech Medical, Racine, WI). High-resolution T1-weighted MRI was acquired from each patient prior to implantation; CT scans were acquired post-implantation. Electrode coordinates obtained from CT were co-registered with each patient's MRI and overlaid onto their reconstructed cortical surface using the method described in Dykstra et al. (2012). Three patients were excluded from the analysis based on poor behavioral performance (false-alarm rates > 0.2 and/or d-prime values < 1). Another patient was excluded based on the fact that the electrodes covered only a small portion of the anterior temporal lobe (and which did not include the *p*STG, the most relevant area for our purposes) where we did not observe robust auditory evoked responses. Thus, we present the results of the remaining patient, whose behavioral performance was well within the normal range for informational masking tasks [which are known to have large intersubject variability (Kidd et al., 2008)], as a case study, with clear recognition that replication is needed (either by us or others) before the findings can be strongly generalized.

The patient whose data we report here was male, aged 31 years at the time of the experiment. Although no audiometry or other audiological testing was conducted, the patient did not report a history of hearing problems. Furthermore, cognitive testing indicated that he was in the average to low average range (though low average for certain auditory memory tests). Analysis of the patient's seizures during invasive monitoring strongly suggested that his epileptogenic focus was in the left medial temporal lobe, a conclusion that was further supported by preoperative MRI and post-operative pathology showing mesial temporal sclerosis in the left hippocampus. The only other abnormality that is potentially relevant for the data presented here was the presence of thickening and increased complexity of the left peri-Sylvian region, consistent with polymicrogyria. However, the patient was able to perform the task and certainly showed activity in the first area where we'd expect it based on prior work (posterolateral temporal cortex).

### Stimuli and procedure

Stimuli were 7.2 s sequences of random pure tones (masker) presented either alone (1/3 of trials) or with an added rhythmic target stream (2/3 of trials; Figure 1A; Supplemental Audio Files 1–4). The subject's task was, first, to indicate by button press the moment at which they began to perceive the target stream and, second, subsequently attend the stream. Every tone in the sequence was 100 ms in duration including 10 ms raised cosine on and off ramps, and was chosen from equally-spaced (logarithmically) frequency bands between 0.239 and 5 kHz. The masker was comprised of tones placed randomly in time and frequency within each band with an average within-band onset asynchrony (SOA) of 800 ms (range: 100–1500 ms). Within each band, the exact frequency of any given tone was within an estimated equivalent rectangular bandwidth (ERB), where ERB = 24.7^*^(4.37^*^fc + 1), where fc is the center frequency of a given band, in kHz. When present, the target stream was comprised of eight identical tones (with one of six frequencies: 0.489, 0.699, 1, 1.430, 2.045, or 2.924 kHz) with an SOA of 800 ms that always began 800 ms after the first potential onset of the masker. Note that this target uncertainly—i.e., the fact that the patient did not know which of the six potential target frequencies was actually the target on any given trial—is one of the primary factors that make the targets difficult to identify in this setup, and a hallmark of informational masking (along with target-masker perceptual similarity). Had the same frequency been used on every trial, we suspect that the targets would have been quite readily identified. Finally, in order to mitigate energetic masking of the target stream, two bands on each side of the target stream were omitted from the masker for trials both with and without the target stream being present.

Sound files were generated in MATLAB (*The Mathworks Inc*., *Natick, MA*) and converted to analog waveforms by the on-board sound card of a laptop computer equipped with Presentation software (*Neurobehavioral Systems, Albany, CA*). Stimuli were delivered to participants via Etymotic ER-2 insert earphones (*Etymotic Research, Inc*., *Elk Grove Village, IL*) binaurally at a comfortable listening level. Participants indicated their detection of target-tone streams via a USB button device (*Cedrus Corporation, San Pedro, CA*). Participants were informed of the fact that the target stream would not be present on every trial but were not told the probability of its occurrence. The start of a new sequence began, on average, 1600 ms after the preceding sequence's termination.

Each experiment was divided into blocks. In each block, 36 target+masker (T+M; Figure 1A, upper panel) stimuli and 18 masker-only (M; Figure 1A, lower panel) stimuli were presented, followed by 18 presentations of control stimuli which were comprised solely of target streams (T). Per block, this yielded 6 repetitions of a T+M condition for each target frequency with each having a different random masker stream, three repetitions of each masker-only condition (where each condition was defined by the frequency of the target stream had it been present), and three repetitions of each T condition. All data shown here used a target-to-masker level ratio of 0 dB (i.e., target tones were the same level as individual masker tones). Note that target+masker and masker trials were randomly interspersed, making it impossible for the patients to know when or when not to expect the presentation of a possible target.

### Data acquisition and analysis

iEEG data from 76 sub-dural electrodes (an 8 × 8 64-contact grid placed on the lateral surface as well as three 4-contact strips on inferior temporal and inferior frontal cortex; Figure S1 in Supplemental Material) were acquired with standard clinical EEG monitoring equipment (XLTEK, Natus Medical Inc., San Carlos, CA) at a sampling rate of 500 Hz. All data were referenced to an inverted intracranial electrode facing the inner skull table remote from the electrodes of interest.

iEEG data were bandpass filtered between 1 and 190 Hz and notch filtered at 60 Hz and its harmonics using zero-phase shift IIR filters. Independent component analysis using the runica algorithm (Bell and Sejnowski, 1995) in EEGLAB (Delorme and Makeig, 2004) was performed on the “raw” data. Components dominated by large artifacts were identified by inspection and projected out of the data.

The iEEG was epoched relative to the onset of individual tones within the target stream (for T+M trials) and, as a control condition derived from the masker-only trials, to the onset of “virtual” target tones, which were time-locked to the onset of target-tone positions, had the target tones been present. For comparison, we also epoched the iEEG relative to the onset of individual tones in the target-only (T) condition. Epoched waveforms were baseline corrected to the 100 ms preceding tone onset (in T+M and T conditions) and to the 100 ms preceding virtual target tone onset (in M conditions). Because the epochs in the masker-only condition are time-locked relative to a non-existent tone onset (virtual targets) and essentially random with respect to any physical stimulus (in this case individual masker tones), the evoked response should average to zero (Gutschalk et al., 2008). This condition was constructed in order to ensure that the presence of such a large number of masker tones (which had random onsets with respect to the tones of interest) did not significantly contribute to the averaged evoked responses in the other conditions. Epochs containing large epileptiform artifacts (5.3% of all epochs) were rejected by visual inspection. Epochs in the T+M condition were binned according to whether or not the target tones were detected by the listener. An individual target tone was defined as “detected” if it fell after the participant indicated by button press that they perceived the target stream. Furthermore, because the task was to detect a repeating target tone (and to do so as quickly as possible), the two individual target tones that preceded a button press were also placed in the “detected” bin. All remaining tones were placed in the “undetected” bin. Note, that while it is possible that patients may have waited to press the button until they heard more than two target tones, this would actually have the effect of biasing our neural results in favor of the null hypothesis of no difference between detected and undetected targets, as some detected targets would likely be placed in the undetected bin.

For the topographic voltage and power maps shown in **Figures 3, 4**, respectively, the data were mapped via in-house software (Dykstra et al., 2012) that was based on the location-on-cortex toolbox, written in MATLAB (Miller et al., 2007). An important parameter one must specify in producing maps using this package is the Gaussian Spreading Parameter, which controls the width of the Gaussian kernel over which activity from an individual electrode is spread. If the parameter is set to zero, then the only vertex that receives any color is that to which an individual electrode is nearest. At the other extreme, if the parameter is set too high, then it's difficult to appreciate foci of activity. We have chosen the parameter such that activity from individual electrodes is still readily visible (as local peaks) while simultaneously smoothing activity between electrodes showing the same activity.

### Statistical analysis

A modified version of the cluster-based, non-parametric statistical procedure outlined previously (Maris and Oostenveld, 2007) was used to test for effects of target detection on target locked EP amplitude. Unpaired *t*-tests were used as the sample-level (i.e., individual time points within a single channel) statistic. Contiguous, statistically-significant samples (defined as *p* < 0.05) within a single electrode were used to define the cluster-level statistic, which was computed by summing the sample-level statistics within a cluster. Statistical significance at the cluster level was determined by computing a Monte Carlo estimate of the permutation distribution of cluster statistics using 1000 re-samples of the original data (Ernst, 2004). Within a single electrode, a cluster was taken to be significant if it fell outside the 95% confidence interval of the permutation distribution for that electrode. The determination of significant clusters was performed independently for each electrode. This method controls the overall false alarm rate within an electrode across time points; no correction for multiple comparisons was performed across electrodes. In order to control for possible confounds of target-tone frequency in the “detected” vs. “undetected” comparison, some target tones at each target-tone frequency were thrown out, making the number of tones in the “detected” and “undetected” bins equal at each frequency.

For the topographic maps shown in **Figures 3, 4**, the only panels that are statistically thresholded based on hypothesis testing are those of the subtraction conditions (i.e., isolated targets minus masker-alone or detected targets minus undetected targets), where any electrode that did not show a significant effect between the two conditions of interest was explicitly set to zero so that it would fall into the gray area of the color map. For the remaining individual conditions (i.e., the top eight panels of **Figure 3** and the top four panels of **Figure 4**), the only “thresholding” is that due to the color map itself, though they are essentially not thresholded given the high resolution of the color map (2^15^ elements). That is, any values that fell < 0.01% of the maximum value of the color mapping, either positive or negative (2^15^-index map, 2^14^-indices on either side of 0), were assigned to gray.

### High-gamma power

Waveforms of high-gamma power were constructed by (i) band-pass filtering between 70 and 190 Hz using zero-phase shift IIR filters, (ii) performing a Hilbert transform, and (iii) squaring the absolute value of the resultant Hilbert-transformed waveforms. This yielded high-gamma power waveforms with the same temporal resolution as the evoked potentials (2 ms). The waveforms were then baseline-corrected in the same manner as the evoked potentials—by subtracting the mean power in each trial averaged over the 100 ms preceding target-tone onset. Finally, the gamma-power waveforms were low-pass filtered at 20 Hz. The same statistical procedures described above for evoked potentials were applied to the high-gamma waveforms.

## Results

### Behavior

Spectrograms of two stimulus examples used in our study as well as behavioral results are shown in Figure 1A. Hit rates for detecting regularly repeating target tones (with a stimulus-onset asynchrony, or SOA, of 800 ms) increased throughout the presentation of the stimulus sequence and plateaued near 50% (Figure 1B). Though false-alarm rates also increased with time since stimulus onset, they remained low overall and never exceeded 20%, resulting in d-prime values that plateaued between 1 and 1.5 (Figure 1C).

### Evoked potentials

Evoked responses were binned and averaged for each of four stimulus/perceptual conditions: (i) target tones presented in isolation, (ii) masker tones presented in isolation, time-locked to the onset of virtual target tones, (iii) detected, and (iv) undetected target tones in target+masker (T+M) sequences. The responses to target tones alone and masker tones alone served as templates with which to compare the responses to detected and undetected target tones presented during T+M conditions.

**Figure 2:**
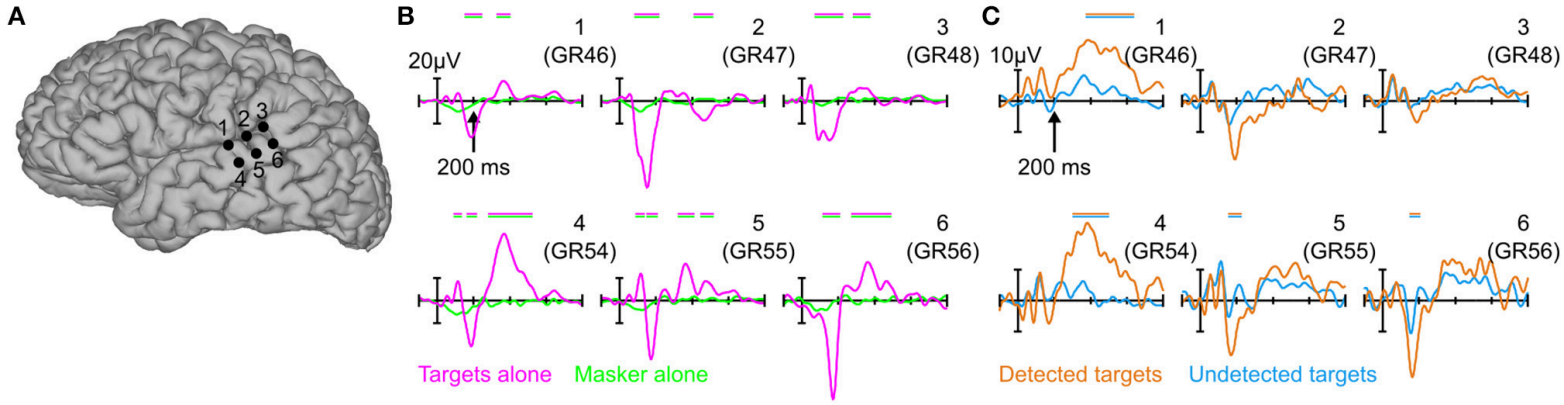
**Evoked potentials over the *p*STG**. **(A)** 3D cortical reconstruction of the patient's brain showing six individual electrode sites over *p*STG for which evoked potentials are plotted in **(B,C)**. **(B)** Evoked potentials in response to the targets-alone (magenta) and masker-alone (green) conditions. magenta/green horizontal bars above the evoked potentials indicates statistically significant differences between the two waveforms in each of the six panels. Note that positive is plotted upwards. **(C)** Same as in **(B)**, but for detected (orange) and undetected (blue) targets in the context of the random multi-tone masker. Orange/blue bars indicate statistically significant differences between the two waveforms in each of the six panels.

Figure 2 shows averaged evoked responses to each condition from six electrode sites over the *p*STG (see also Figures S2, S3 in the Supplemental Material for evoked responses from all 64 contacts on the grid). As expected, since the masker-only epochs were time-locked to virtual tone onsets (effectively random time-locking with respect to individual masker tones, see Section Materials and Methods), the averaged response for these epochs was flat (Figure 2B, green traces), indicating that the presence of the masker tones alone does not significantly contribute to the evoked responses in the other conditions. In contrast, the averaged evoked responses to targets presented in isolation were robust and showed a stereotypical pattern over the *p*STG characterized first by a large surface-negative response (peaking at 162 ms) followed by a broad long-latency positivity peaking between 300 and 600 ms (Figure 2B, magenta traces). Qualitatively, both these components can be seen for detected targets in the presence of the multi-tone masker (Figure 2C, orange traces), with perhaps minor differences in response latency, size, and topography. The response to undetected targets (Figure 2C, blue traces) was relatively flat by comparison, similar to the response for the masker-alone condition, although some earlier components may be equally present for detected and undetected targets, particularly the positive-going deflections before ~150 ms.

**Figure 3:**
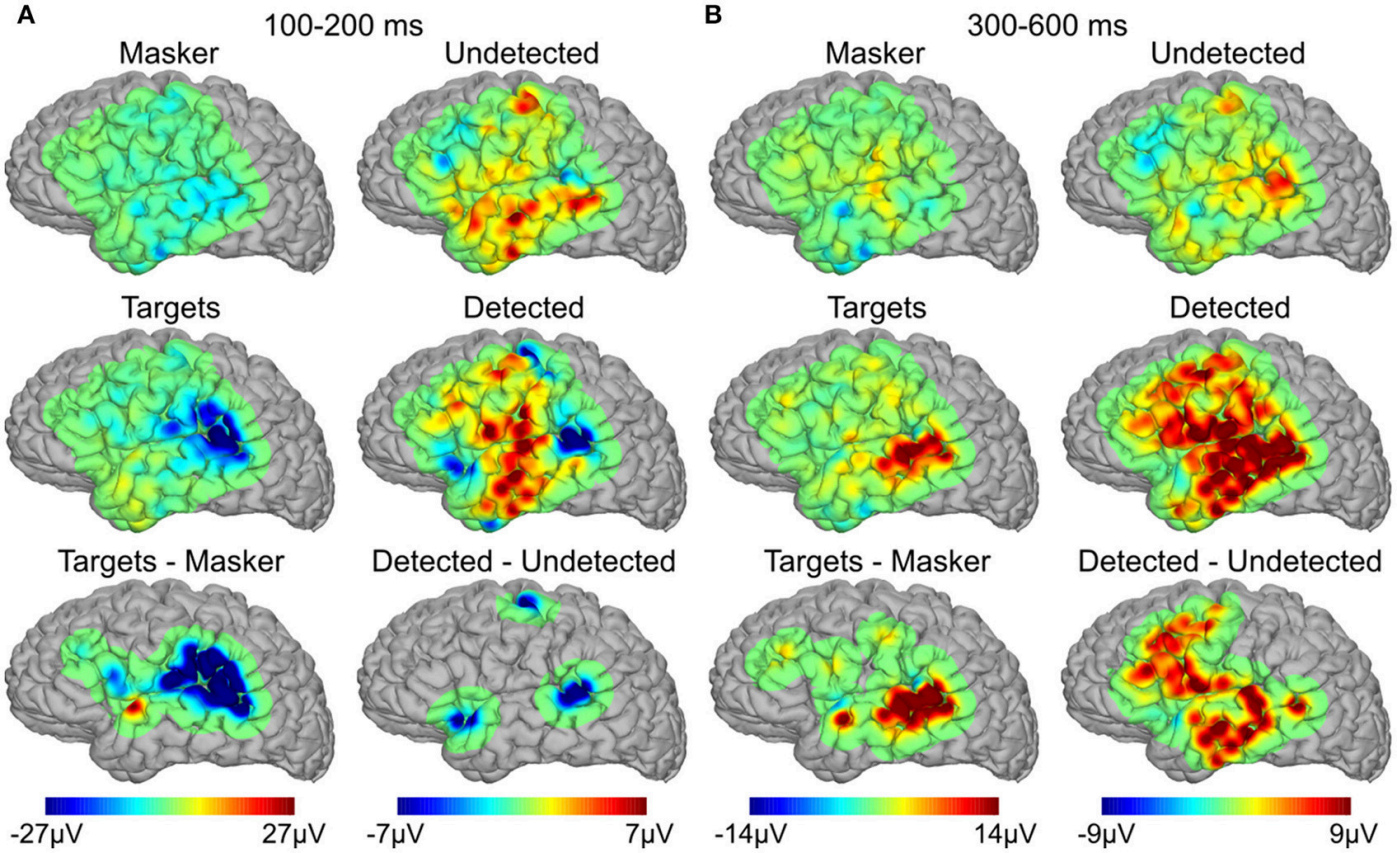
**Spatial topographies of the evoked responses**. **(A)** Average topography of the evoked responses between 100 and 200 ms for targets alone, masker alone, and their subtraction (left three panels), and detected targets, undetected targets, and their subtraction (right three panels). **(B)** Same as in **(A)**, between 300 and 600 ms.

Topographical maps of averaged potentials are shown in Figure 3 for two latency ranges, 100–200 ms (Figure 3A) and 300–600 ms (Figure 3B) and each of the four behavioral conditions (top two rows) in addition to their subtraction (bottom row). In all cases, color values reflect the mean signal within the given latency range. All panels in the bottom row have been thresholded to show only the activity near electrode sites which showed statistically-significant differences between the corresponding conditions (either targets alone vs. masker alone or detected vs. undetected targets) in the corresponding latency range. The primary differentiation of detected vs. undetected targets in the earlier latency range is over the posterior auditory cortex (Figure 3A, right lower-most panel). This was similar to the differentiation seen between targets-alone and masker-alone (Figure 3A, left lower-most panel). In contrast, the longer-latency response that differentiated between detected and undetected targets was present in much more widespread brain areas than for targets-alone vs. masker-alone in the same latency range (Figure 3B, compare right lower-most panel vs. left lower-most panel), particularly ventrolateral frontal and lateral temporal cortices. In the contrast of isolated vs. virtual targets (i.e., targets alone vs. masker alone), activity was also observed in the posterior aspects of the superior and middle temporal gyrus, but not in frontal cortex or more anteroventral portions of the temporal lobe.

### High-gamma activity

**Figure 4:**
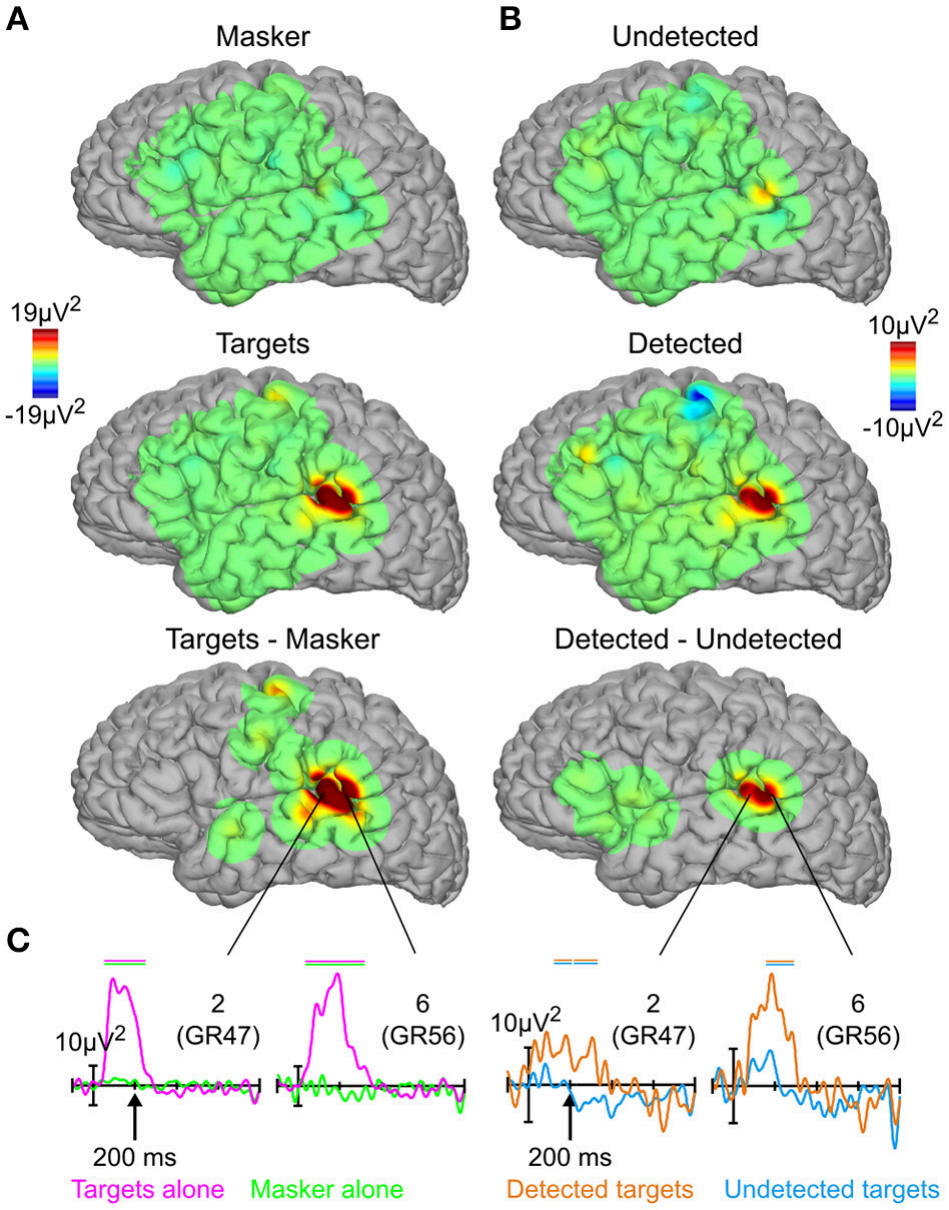
**High-gamma activity**. **(A)** Topographies of high-gamma activity between 50 and 250 ms for targets alone, masker alone, and their subtraction. **(B)** Same as in **(A)**, for detected targets, undetected targets, and their subtraction. **(C)** High-gamma responses for targets alone (magenta), masker alone (green), detected targets (orange), and undetected targets (blue) from the two sites showing the largest high-gamma response. Horizontal bars above the high-gamma waveforms indicate statistically significant differences; positive plotted upwards. The numbers in the upper right-hand corner of each panel refer to the electrode sites from Figure 2.

Figures 4A,B show topographical plots of mean HGA between 50 and 250 ms for targets and masker-alone, detected, and undetected targets, and their respective subtractions, where the subtraction maps have been thresholded for statistical significance. The HGA was much more focal than the evoked responses, confined mostly to the posterior auditory cortex near the sites which showed maximal evoked responses between 100 and 200 ms (cf. Figure 3A). Figure 4C shows the full time courses of the two electrodes which showed the largest high-gamma response for both targets vs. masker alone (two left-most panels) and detected vs. undetected targets (two right-most panels). As with the evoked responses, the responses to masker-alone, and undetected-target conditions are relatively flat, more so in the masker-alone condition. In contrast, targets-alone and detected-target conditions elicited robust responses peaking between 50 and 250 ms, roughly the same latency range as the corresponding (early) evoked responses, though the onset of the high-gamma effect may be slightly earlier.

## Discussion

Utilizing direct cortical recordings in a human neurosurgical patient, the present study observed robust correlates of auditory perceptual awareness in widespread brain areas, including early (50–250 ms) evoked responses and HGA in posterolateral auditory cortex as well as a broad long-latency (300–600 ms) potential in ventrolateral frontal and lateral temporal cortex. This represents direct evidence that both auditory HGA and slow, P3b-like potentials in supra-modal areas can covary with auditory perceptual awareness in complex listening situations, and extends previous work showing such correlates in early evoked responses arising from auditory cortex. However, the extent to which these responses reflect conscious perception, itself, as opposed to something else (e.g., selective attention, pre- or post-perceptual processing) remains unclear and is discussed below.

### Early responses in auditory cortex

Despite their similar latency, it is unclear whether the early evoked responses we measured over *p*STG are akin to those from previous MEG studies [the sources of which were localized to the posterior superior temporal plane (Gutschalk et al., 2008; Wiegand and Gutschalk, 2012; Dykstra and Gutschalk, 2015)] given that (i) we did not observe polarity reversals across the lateral fissure (cf. Figure 3A), (ii) the locus of response we measured was more posterior than the dipole locations reported by that study, and (iii) the fact that intracranial EEG is much more sensitive to proximal vs. distal sources. Thus, the early evoked responses we measured may be generated by radially oriented *p*STG sources just underneath the electrode (sources to which MEG would not be sensitive), perhaps related to the N150 component (also known as the negative-going portion of the T-complex) of the auditory evoked potential (Wolpaw and Penry, 1975; Celesia, 1976; Scherg et al., 1989). An alternative interpretation is that they arise from a diffuse source in posterior auditory cortex that only sometimes extends to the lateral portion of the *p*STG. This could potentially be examined non-invasively using EEG, which is sensitive to the presence of radial sources (Picton et al., 1999), or with penetrating depth electrodes in certain neurosurgical patients (Nourski and Howard, 2015).

The present study also observed early HGA that covaried with auditory perceptual awareness in some of the same sites that showed robust early evoked potentials. However, unlike the evoked responses, the HGA was highly focal, confined to just two electrodes over the *p*STG [the posterolateral superior temporal area described previously (Howard et al., 2000)], consistent with recent intracranial findings of auditory target detection (Nourski et al., 2015). With the exception of sparse, weak effects elsewhere, this activity did not extend into other brain areas or longer latencies. Intracranial HGA, especially when compared with intracranial evoked potentials, is consistently found to be highly focal, including in the context of auditory tasks (Edwards et al., 2005; Dykstra et al., 2011), and is thought to reflect active neural processes that produce high-frequency synchronized responses in the immediate vicinity of the electrode (Lachaux et al., 2012). This idea is consistent with recent studies of visual perceptual awareness in which perceived visual stimuli were associated with much greater HGA than unperceived stimuli (Fisch et al., 2009; Gaillard et al., 2009; but see Aru et al., 2012a; Pitts et al., 2014b).

This early activation of auditory cortex for detected targets in the context of the multi-tone masker may reflect either recurrent activity posited by some to reflect perceptual awareness (Lamme, 2006), or processes that are antecedent to it. However, one factor that is difficult to rule out is selective attention (Snyder et al., 2012), which is known to modulate auditory evoked responses in the same latency range as the detected/undetected effects shown here (Hillyard et al., 1973; Hansen and Hillyard, 1980; Woldorff et al., 1993; Gutschalk et al., 2008; Ahveninen et al., 2011; Gutschalk and Dykstra, 2014; Lee et al., 2014). This is particularly true for tones coming after listeners indicated their awareness of the target stream, and remains to be clarified by future studies capable of independently manipulating auditory attention and perceptual awareness.

### Widespread long-latency responses

In addition to early activity in *p*STG sites, detected targets also elicited late (~300–600 ms), distributed responses over posterior auditory, ventrolateral frontal, and lateral temporal cortex, with primarily the auditory component elicited by targets presented in isolation, with perhaps some extension into the superior temporal sulcus. To our knowledge, this is first direct report of such distributed activity during this task (but see Giani et al., 2015), and previous work may have failed to detect it due to the use of dipole models chosen to focus on activity arising from auditory cortex. Alternatively, given that the activity is spread over several gyri/sulci (particularly in frontal cortex), a substantial portion of this activity may cancel at sites distant from the generators, such as is the case with M/EEG (Ahlfors et al., 2010). The *p*STG source at the same latency may be difficult to observe with MEG due to its insensitivity to radial sources.

The response to detected targets in the presence of the multi-tone masker resembles the P3b in that it was late, broad, prominent in ventrolateral frontal, and lateral temporal cortex (anterior and ventral to the focus over *p*STG—cf. two lower-most panels of Figure 3B), and only present when the subjects were engaged in an active task (Halgren et al., 1998; Linden, 2005; Halgren, 2008). This is notable due to the fact that the P3b has often been taken as a marker of conscious perception (Dehaene and Changeux, 2011). However, the fact that the supra-modal components of this response were only seen for detected targets during informational masking and not for targets presented in isolation (which are presumably perceived) suggests that they likely reflect something else. One possibility is post-perceptual processing associated with task relevance of the target stream during informational masking. This interpretation would be consistent with previous studies showing that both the P3b and frontal activity (as measured with unit recordings or fMRI) depend strongly on task relevance and context (Hillyard et al., 1971; Fritz et al., 2010; Melloni et al., 2011; Pitts et al., 2012, 2014b), and highlights the importance of employing passive as well as active paradigms in examining the neural correlates of consciousness (Deouell, 2002; Aru et al., 2012b; Pitts et al., 2014a; Tsuchiya et al., 2015).

However, even if this activity doesn't reflect conscious perception for isolated stimuli, we cannot rule out the possibility that it might reflect conscious perception for the masker-embedded target streams used here. More generally, such activity, which may also reflect sources of attentional enhancement (Corbetta and Shulman, 2002; Fritz et al., 2007; Gutschalk and Dykstra, 2015) that can help bring stimuli into consciousness (Dehaene et al., 2006; Snyder et al., 2012), might be necessary to perceive target stimuli in noise or under conditions of high perceptual load (Gutschalk and Dykstra, 2014; Lavie et al., 2014). This would be consistent with recent studies showing enhanced ventrolateral frontal activity during attention toward acoustic target stimuli that were difficult to perceive (Hill and Miller, 2010; Wild et al., 2012; Zion Golumbic et al., 2013).

Going forward, one way to address the question of whether the late responses we observed embody conscious perception would be to examine whether they (or any other responses we observed) differ depending on tone position within the target-stream sequence. While this is something that has been observed previously for earlier evoked responses elicited by detected targets in the context of the multi-tone masker (Gutschalk et al., 2008) and for HGA in simpler acoustic sequences (Edwards et al., 2005; Eliades et al., 2014), we simply do not have the SNR to be able to address that question here. Finally, whether other areas (auditory or otherwise) not sampled by the present study might show activity associated with conscious perception of such masker-embedded target streams remains to be examined by future studies.

## Author contributions

AD, SC, and EH designed the research. AD, EE, and SC performed the research. AG contributed MATLAB scripts for stimulus generation. AD, SC, EH, and AG wrote the paper.

## Funding

This work was supported by NIDCD grant T32 DC00038, NIBIB grant T32 EB001680, and an Amelia Peabody Charitable Trust grant to AD, NIH grant NS18741 to EH, and NINDS grant NS062092 to SC.

### Conflict of interest statement

The authors declare that the research was conducted in the absence of any commercial or financial relationships that could be construed as a potential conflict of interest.

## References

[B1] Ahlfors S. P. Han J. Lin F.-H. Witzel T. Belliveau J. W. Hämäläinen M. S.. (2010). Cancellation of EEG and MEG signals generated by extended and distributed sources. Hum. Brain Mapp. 31, 140–149. 10.1002/hbm.20851 19639553 PMC2797557

[B2] Ahveninen J. Hämäläinen M. Jääskeläinen I. P. Ahlfors S. P. Huang S. Lin F.-H.. (2011). Attention-driven auditory cortex short-term plasticity helps segregate relevant sounds from noise. Proc. Natl. Acad. Sci. U.S.A. 108, 4182–4187. 10.1073/pnas.1016134108 21368107 PMC3053977

[B3] Aru J. Axmacher N. Do Lam A. T. A. Fell J. Elger C. E. Singer W.. (2012a). Local category-specific gamma band responses in the visual cortex do not reflect conscious perception. J. Neurosci. 32, 14909–14914. 10.1523/JNEUROSCI.2051-12.2012 23100413 PMC6704831

[B4] Aru J. Bachmann T. Singer W. Melloni L. (2012b). Distilling the neural correlates of consciousness. Neurosci. Biobehav. Rev. 36, 737–746. 10.1016/j.neubiorev.2011.12.003 22192881

[B5] Bell A. J. Sejnowski T. J. (1995). An information-maximization approach to blind separation and blind deconvolution. Neural Comput. 7, 1129–1159. 10.1162/neco.1995.7.6.1129 7584893

[B6] Cariani P. A. Micheyl C. (2012). Towards a theory of information processing in the auditory cortex, in The Human Auditory Cortex, eds Poeppel D. Overath T. Popper A. Fay R. R.(New York, NY: Springer), 351–390.

[B7] Celesia G. G. (1976). Organization of auditory cortical areas in man. Brain 99, 403–414. 10.1093/brain/99.3.403 1000279

[B8] Corbetta M. Shulman G. L. (2002). Control of goal-directed and stimulus-driven attention in the brain. Nat. Rev. Neurosci. 3, 215–229. 10.1038/nrn755 11994752

[B9] Dehaene S. Changeux J.-P. (2011). Experimental and theoretical approaches to conscious processing. Neuron 70, 200–227. 10.1016/j.neuron.2011.03.018 21521609

[B10] Dehaene S. Changeux J.-P. Naccache L. Sackur J. Sergent C. (2006). Conscious, preconscious, and subliminal processing: a testable taxonomy. Trends Cogn. Sci. 10, 204–211. 10.1016/j.tics.2006.03.007 16603406

[B11] Delgutte B. (1990). Physiological mechanisms of psychophysical masking: observations from auditory-nerve fibers. J. Acoust. Soc. Am. 87, 791–809. 10.1121/1.398891 2307776

[B12] Delorme A. Makeig S. (2004). EEGLAB: an open source toolbox for analysis of single-trial EEG dynamics including independent component analysis. J. Neurosci. Methods 134, 9–21. 10.1016/j.jneumeth.2003.10.009 15102499

[B13] Deouell L. Y. (2002). Pre-requisites for conscious awareness: clues from electrophysiological and behavioral studies of unilateral neglect patients. Conscious. Cogn. 11, 546–567. 10.1016/S1053-8100(02)00024-7 12470622

[B14] Dykstra A. R. Chan A. M. Quinn B. T. Zepeda R. Keller C. J. Cormier J.. (2012). Individualized localization and cortical surface-based registration of intracranial electrodes. Neuroimage 59, 3563–3570. 10.1016/j.neuroimage.2011.11.046 22155045 PMC3288767

[B15] Dykstra A. R. Gutschalk A. (2015). Does the mismatch negativity operate on a consciously accessible memory trace? Sci. Adv. 1:e1500677. 10.1126/sciadv.1500677 26702432 PMC4681331

[B16] Dykstra A. R. Halgren E. Thesen T. Carlson C. E. Doyle W. Madsen J. R.. (2011). Widespread brain areas engaged during a classical auditory streaming task revealed by intracranial EEG. Front. Hum. Neurosci. 5:74. 10.3389/fnhum.2011.00074 21886615 PMC3154443

[B17] Edwards E. Soltani M. Deouell L. Y. Berger M. S. Knight R. T. (2005). High gamma activity in response to deviant auditory stimuli recorded directly from human cortex. J. Neurophysiol. 94, 4269–4280. 10.1152/jn.00324.2005 16093343

[B18] Elhilali M. Xiang J. Shamma S. A. Simon J. Z. (2009). Interaction between attention and bottom-up saliency mediates the representation of foreground and background in an auditory scene. PLoS Biol. 7:e1000129. 10.1371/journal.pbio.1000129 19529760 PMC2690434

[B19] Eliades S. J. Crone N. E. Anderson W. S. Ramadoss D. Lenz F. A. Boatman-Reich D. (2014). Adaptation of high-gamma responses in human auditory association cortex. J. Neurophysiol. 112, 2147–2163. 10.1152/jn.00207.2014 25122702 PMC4274926

[B20] Ernst M. D. (2004). Permutation methods: a basis for exact inference. Stat. Sci. 19, 676–685. 10.1214/088342304000000396

[B21] Fisch L. Privman E. Ramot M. Harel M. Nir Y. Kipervasser S.. (2009). Neural “ignition”: enhanced activation linked to perceptual awareness in human ventral stream visual cortex. Neuron 64, 562–574. 10.1016/j.neuron.2009.11.001 19945397 PMC2854160

[B22] Fritz J. B. David S. V. Radtke-Schuller S. Yin P. Shamma S. A. (2010). Adaptive, behaviorally gated, persistent encoding of task-relevant auditory information in ferret frontal cortex. Nat. Neurosci. 13, 1011–1019. 10.1038/nn.2598 20622871 PMC2921886

[B23] Fritz J. B. Elhilali M. David S. V. Shamma S. A. (2007). Auditory attention–focusing the searchlight on sound. Curr. Opin. Neurobiol. 17, 437–455. 10.1016/j.conb.2007.07.011 17714933

[B24] Gaillard R. Dehaene S. Adam C. Clémenceau S. Hasboun D. Baulac M.. (2009). Converging intracranial markers of conscious access. PLoS Biol. 7:e61. 10.1371/journal.pbio.1000061 19296722 PMC2656551

[B25] Giani A. S. Belardinelli P. Ortiz E. Kleiner M. Noppeney U. (2015). Detecting tones in complex auditory scenes. Neuroimage 122, 203–213. 10.1016/j.neuroimage.2015.07.001 26244276

[B26] Gutschalk A. Dykstra A. (2015). Auditory neglect and related disorders. Handb. Clin. Neurol. 129, 557–571. 10.1016/B978-0-444-62630-1.00031-7 25726290

[B27] Gutschalk A. Dykstra A. R. (2014). Functional imaging of auditory scene analysis. Hear. Res. 307, 98–110. 10.1016/j.heares.2013.08.003 23968821

[B28] Gutschalk A. Micheyl C. Oxenham A. J. (2008). Neural correlates of auditory perceptual awareness under informational masking. PLoS Biol. 6:e138. 10.1371/journal.pbio.0060138 18547141 PMC2422852

[B29] Halgren E. (2004). How can intracranial recordings assist MEG source localization? Neurol. Clin. Neurophysiol. 2004:86. 16012657

[B30] Halgren E. (2008). Brain states measured as the P3, in Event-Related Potentials in Patients with Epilepsy: From Current State to Future Prospects, eds Inoue Y. Ikeda A.(Paris: John Libbey Eurotext), 11–26.

[B31] Halgren E. Marinkovic K. Chauvel P. (1998). Generators of the late cognitive potentials in auditory and visual oddball tasks. Electroencephalogr. Clin. Neurophysiol. 106, 156–164. 10.1016/S0013-4694(97)00119-3 9741777

[B32] Hansen J. C. Hillyard S. A. (1980). Endogenous brain potentials associated with selective auditory attention. Electroencephalogr. Clin. Neurophysiol. 49, 277–290. 10.1016/0013-4694(80)90222-9 6158404

[B33] Hill K. T. Miller L. M. (2010). Auditory attentional control and selection during cocktail party listening. Cereb. Cortex 20, 583–590. 10.1093/cercor/bhp124 19574393 PMC2820699

[B34] Hillyard S. A. Hink R. F. Schwent V. L. Picton T. W. (1973). Electrical signs of selective attention in the human brain. Science 182, 177–180. 10.1126/science.182.4108.177 4730062

[B35] Hillyard S. A. Squires K. C. Bauer J. W. Lindsay P. H. (1971). Evoked potential correlates of auditory signal detection. Science 172, 1357–1360. 10.1126/science.172.3990.1357 5580218

[B36] Howard M. A. Volkov I. O. Mirsky R. Garell P. C. Noh M. D. Granner M.. (2000). Auditory cortex on the human posterior superior temporal gyrus. J. Comp. Neurol. 416, 79–92. 10.1002/(SICI)1096-9861(20000103)416:1<79::AID-CNE6>3.0.CO;2-2 10578103

[B37] Irimia A. Van Horn J. D. Halgren E. (2012). Source cancellation profiles of electroencephalography and magnetoencephalography. Neuroimage 59, 2464–2474. 10.1016/j.neuroimage.2011.08.104 21959078 PMC3254784

[B38] Kidd G. Mason C. R. Richards V. M. Gallun F. J. Durlach N. I. (2008). Informational masking, in Auditory Perception of Sound Sources, eds Yost W. A. Popper A. N. Fay R. R.(New York, NY: Springer), 143–190.

[B39] Koch C. Massimini M. Boly M. Tononi G. (2016). Neural correlates of consciousness: progress and problems. Nat. Rev. Neurosci. 17, 307–321. 10.1038/nrn.2016.22 27094080

[B40] Lachaux J.-P. Axmacher N. Mormann F. Halgren E. Crone N. E. (2012). High-frequency neural activity and human cognition: past, present and possible future of intracranial EEG research. Prog. Neurobiol. 98, 279–301. 10.1016/j.pneurobio.2012.06.008 22750156 PMC3980670

[B41] Lamme V. A. F. (2006). Towards a true neural stance on consciousness. Trends Cogn. Sci. 10, 494–501. 10.1016/j.tics.2006.09.001 16997611

[B42] Lavie N. Beck D. M. Konstantinou N. (2014). Blinded by the load: attention, awareness and the role of perceptual load. Philos. Trans. R. Soc. Lond. B Biol. Sci. 369:20130205. 10.1098/rstb.2013.0205 24639578 PMC3965161

[B43] Lee A. K. C. Larson E. Maddox R. K. Shinn-Cunningham B. G. (2014). Using neuroimaging to understand the cortical mechanisms of auditory selective attention. Hear. Res. 307, 111–120. 10.1016/j.heares.2013.06.010 23850664 PMC3844039

[B44] Linden D. E. J. (2005). The p300: where in the brain is it produced and what does it tell us? Neuroscientist 11, 563–576. 10.1177/1073858405280524 16282597

[B45] Manning J. R. Jacobs J. Fried I. Kahana M. J. (2009). Broadband shifts in local field potential power spectra are correlated with single-neuron spiking in humans. J. Neurosci. 29, 13613–13620. 10.1523/JNEUROSCI.2041-09.2009 19864573 PMC3001247

[B46] Maris E. Oostenveld R. (2007). Nonparametric statistical testing of EEG- and MEG-data. J. Neurosci. Methods 164, 177–190. 10.1016/j.jneumeth.2007.03.024 17517438

[B47] Melloni L. Schwiedrzik C. M. Müller N. Rodriguez E. Singer W. (2011). Expectations change the signatures and timing of electrophysiological correlates of perceptual awareness. J. Neurosci. 31, 1386–1396. 10.1523/JNEUROSCI.4570-10.2011 21273423 PMC6623627

[B48] Miller K. J. Makeig S. Hebb A. O. Rao R. P. N. denNijs M. Ojemann J. G. (2007). Cortical electrode localization from X-rays and simple mapping for electrocorticographic research: the “Location on Cortex” (LOC) package for MATLAB. J. Neurosci. Methods 162, 303–308. 10.1016/j.jneumeth.2007.01.019 17343918

[B49] Mukamel R. Gelbard H. Arieli A. Hasson U. Fried I. Malach R. (2005). Coupling between neuronal firing, field potentials, and FMRI in human auditory cortex. Science 309, 951–954. 10.1126/science.1110913 16081741

[B50] Neff D. L. Dethlefs T. M. Jesteadt W. (1993). Informational masking for multicomponent maskers with spectral gaps. J. Acoust. Soc. Am. 94, 3112–3126. 10.1121/1.407217 8300950

[B51] Neff D. L. Green D. M. (1987). Masking produced by spectral uncertainty with multicomponent maskers. Percept. Psychophys. 41, 409–415. 10.3758/BF03203033 3601622

[B52] Nourski K. V. Howard M. A. (2015). Invasive recordings in the human auditory cortex, in Handbook of Clinical Neurology, eds Celesia G. G. Hickok G.(Amsterdam: Elsevier), 225–244. 10.1016/B978-0-444-62630-1.00013-5 25726272

[B53] Nourski K. V. Steinschneider M. Oya H. Kawasaki H. Howard M. A. (2015). Modulation of response patterns in human auditory cortex during a target detection task: an intracranial electrophysiology study. Int. J. Psychophysiol. 95, 191–201. 10.1016/j.ijpsycho.2014.03.006 24681353 PMC4430839

[B54] Picton T. W. Alain C. Woods D. L. John M. S. Scherg M. Valdes-Sosa P.. (1999). Intracerebral sources of human auditory-evoked potentials. Audiol. Neurootol. 4, 64–79. 10.1159/000013823 9892757

[B55] Pitts M. A. Martínez A. Hillyard S. A. (2012). Visual processing of contour patterns under conditions of inattentional blindness. J. Cogn. Neurosci. 24, 287–303. 10.1162/jocn_a_00111 21812561

[B56] Pitts M. A. Metzler S. Hillyard S. A. (2014a). Isolating neural correlates of conscious perception from neural correlates of reporting one's perception. Front. Psychol. 5:1078. 10.3389/fpsyg.2014.01078 25339922 PMC4189413

[B57] Pitts M. A. Padwal J. Fennelly D. Martínez A. Hillyard S. A. (2014b). Gamma band activity and the P3 reflect post-perceptual processes, not visual awareness. Neuroimage 101, 337–350. 10.1016/j.neuroimage.2014.07.024 25063731 PMC4169212

[B58] Pollack I. (1975). Auditory informational masking. J. Acoust. Soc. Am. 57, S5 10.1121/1.1995329

[B59] Scherg M. Vajsar J. Picton T. W. (1989). A source analysis of the late human auditory evoked potentials. J. Cogn. Neurosci. 1, 336–355. 10.1162/jocn.1989.1.4.336 23971985

[B60] Snyder J. S. Gregg M. K. Weintraub D. M. Alain C. (2012). Attention, awareness, and the perception of auditory scenes. Front. Psychol. 3:15. 10.3389/fpsyg.2012.00015 22347201 PMC3273855

[B61] Snyder J. S. Yerkes B. D. Pitts M. A. (2015). Testing domain-general theories of perceptual awareness with auditory brain responses. Trends Cogn. Sci. 19, 295–297. 10.1016/j.tics.2015.04.002 25960421

[B62] Squires K. C. Hillyard S. A. Lindsay P. H. (1973). Vertex potentials evoked during auditory signal detection: relation to decision criteria. Percept. Psychophys. 14, 265–272. 10.3758/BF03212388

[B63] Steinschneider M. Fishman Y. I. Arezzo J. C. (2008). Spectrotemporal analysis of evoked and induced electroencephalographic responses in primary auditory cortex (A1) of the awake monkey. Cereb. Cortex 18, 610–625. 10.1093/cercor/bhm094 17586604

[B64] Tsuchiya N. Wilke M. Frässle S. Lamme V. A. F. (2015). No-report paradigms: extracting the true neural correlates of consciousness. Trends Cogn. Sci. 19, 757–770. 10.1016/j.tics.2015.10.002 26585549

[B65] Wiegand K. Gutschalk A. (2012). Correlates of perceptual awareness in human primary auditory cortex revealed by an informational masking experiment. Neuroimage 61, 62–69. 10.1016/j.neuroimage.2012.02.067 22406354

[B66] Wild C. J. Yusuf A. Wilson D. E. Peelle J. E. Davis M. H. Johnsrude I. S. (2012). Effortful listening: the processing of degraded speech depends critically on attention. J. Neurosci. 32, 14010–14021. 10.1523/JNEUROSCI.1528-12.2012 23035108 PMC6704770

[B67] Woldorff M. G. Gallen C. C. Hampson S. A. Hillyard S. A. Pantev C. Sobel D.. (1993). Modulation of early sensory processing in human auditory cortex during auditory selective attention. Proc. Natl. Acad. Sci. U.S.A. 90, 8722–8726. 10.1073/pnas.90.18.8722 8378354 PMC47430

[B68] Wolpaw J. R. Penry J. K. (1975). A temporal component of the auditory evoked response. Electroencephalogr. Clin. Neurophysiol. 39, 609–620. 10.1016/0013-4694(75)90073-5 53139

[B69] Zion Golumbic E. M. Ding N. Bickel S. Lakatos P. Schevon C. A. McKhann G. M.. (2013). Mechanisms underlying selective neuronal tracking of attended speech at a “Cocktail party.” Neuron 77, 980–991. 10.1016/j.neuron.2012.12.037 23473326 PMC3891478

