# Supplementary material for: Neural Correlates of Auditory Perceptual Awareness and Release from Informational Masking Recorded Directly from Human Cortex: A Case Study
Source: Front Neurosci. 2016 Oct 20;10:472. doi: 10.3389/fnins.2016.00472 (PMC5071374; doi:10.3389/fnins.2016.00472)
Supplement: Supplementary file 1 [file DataSheet1.PDF]

Captions for Supplemental Audio Files

Audio 1. Example target+masker (T+M) stimulus. An example of a multi-tone masker stimulus, with a regularly repeating target-tone stream at 1,435 Hz embedded. Quality headphones are recommended. Initially, the target tones can be quite difficult to hear as a separate stream, and are much more readily identified after listening to either a filtered version of the sequence (Audio 2) or the target stream in isolation (Audio 3). This was the stimulus used to obtain the "detected" and "undetected" target responses.

Audio 2. Filtered version of Sound 1. The same stimulus as in Audio 1, but with a narrow band-pass filter applied around the frequency of the target stream (1,435 Hz).

Audio 3. Example targets-alone (T) stimulus. Here, the 1,435 Hz target stream is presented in isolation, making it much easier to identify when embedded in the multi-tone masker (Audio 1). This was the stimulus used to obtain the "targets-alone" responses.

Audio 4. Example of masker-alone (M) stimulus. Same as Audio 1, without the target stream. This was the stimulus used to obtain the "masker-alone" responses.

Supplemental Figures

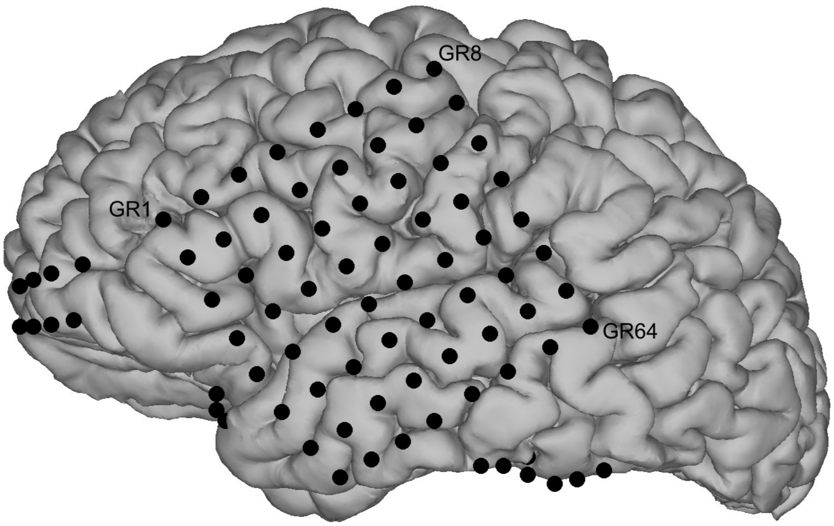

Figure S1. Electrode coverage on the patients cortical surface. Black dots indicates 2.3-mm sub-dural electrocorticography electrodes.

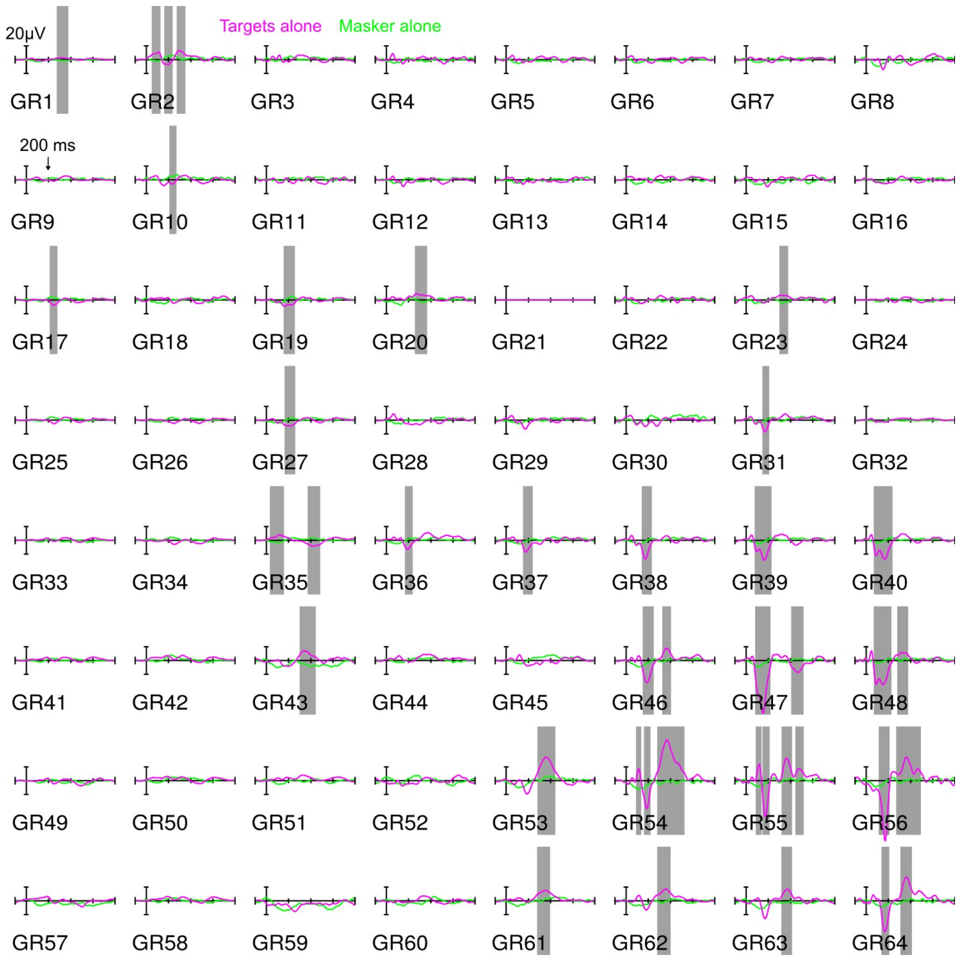

Figure S2. Evoked responses for the control conditions. Targets-only (green) and masker-only (magenta) evoked responses from each of the 64 electrodes comprising the 8x8 grid. Statistically significant differences between the two conditions are indicated by gray shading.

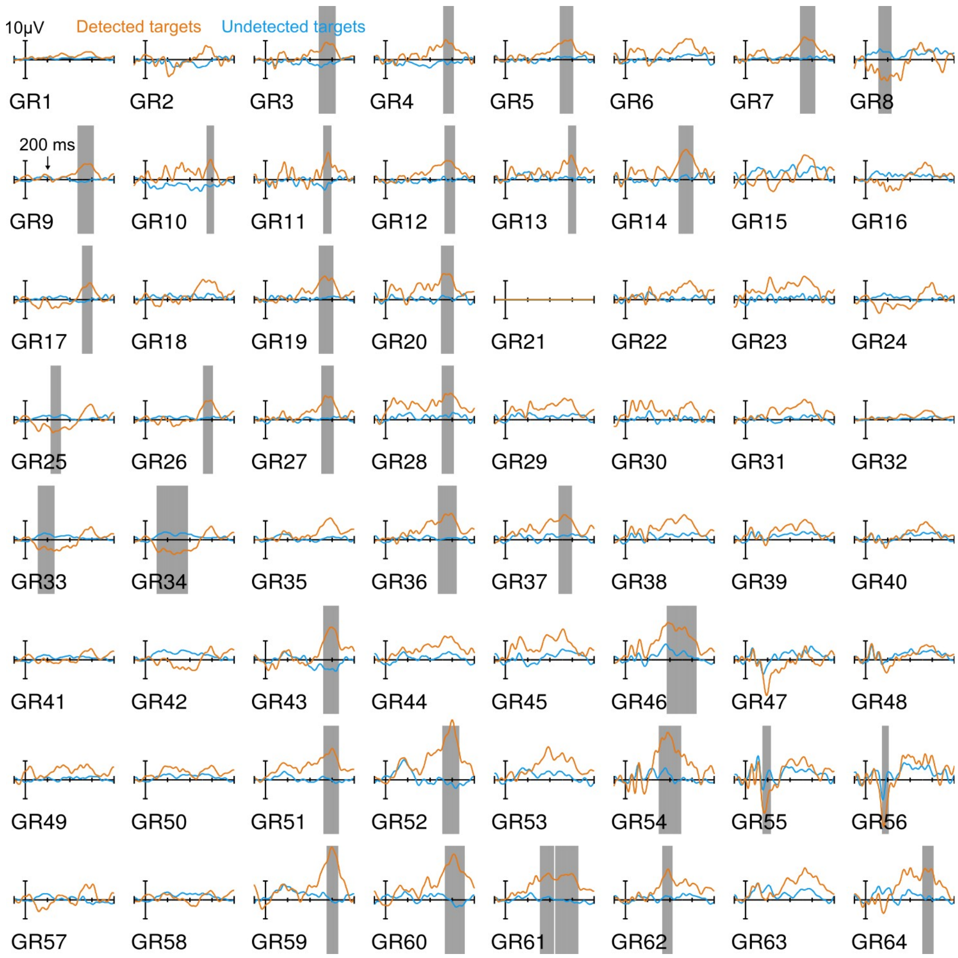

Figure S3. Same as Fig. S2, for detected-target (orange) and undetected-target (blue) responses.
